# Role of Type2 Inflammatory Biomarkers in Chronic Obstructive Pulmonary Disease

**DOI:** 10.3390/jcm9082670

**Published:** 2020-08-18

**Authors:** Keiji Oishi, Kazuto Matsunaga, Toshihiro Shirai, Keita Hirai, Yasuhiro Gon

**Affiliations:** 1 Department of Medicine and Clinical Science, Graduate School of Medicine, Yamaguchi University, Yamaguchi 755-8505, Japan; 2 Department of Respiratory Medicine and Infectious Disease, Graduate School of Medicine, Yamaguchi University, Yamaguchi 755-8505, Japan; kazmatsu@yamaguchi-u.ac.jp; 3 Department of Respiratory Medicine, Shizuoka General Hospital, Shizuoka 420-8527, Japan; toshihiro-shirai@i.shizuoka-pho.jp; 4 Department of Clinical Pharmacology and Genetics, School of Pharmaceutical Sciences, University of Shizuoka, Shizuoka 422-8526, Japan; hiraik@u-shizuoka-ken.ac.jp; 5 Laboratory of Clinical Pharmacogenomics, Shizuoka General Hospital, Shizuoka 420-8527, Japan; 6 Division of Respiratory Medicine, Department of Internal Medicine, Nihon University School of Medicine, Tokyo 173-8601, Japan; gon.yasuhiro@nihon-u.ac.jp

**Keywords:** airway inflammation, asthma-chronic obstructive pulmonary disease overlap, atopy, chitinase-3-like protein 1, eosinophil-derived neurotoxin, eosinophils, fractional exhaled nitric oxide, inhaled corticosteroid response, periostin

## Abstract

Airway inflammation in chronic obstructive pulmonary disease (COPD) is typically thought to be driven by Type1 immune responses, while Type2 inflammation appears to be present in definite proportions in the stable state and during exacerbations. In fact, some COPD patients showed gene expression of Type2 inflammation in the airway, and this subset was associated with the inhaled corticosteroid (ICS) response. Interestingly enough, the relationship between COPD and diseases associated with Type2 inflammation from the perspective of impaired lung development is increasingly highlighted by recent epidemiologic studies on the origin of COPD. Therefore, many researchers have shown an interest in the prevalence and the role of existent Type2 biomarkers such as sputum and blood eosinophils, exhaled nitric oxide fraction, and atopy, not only in asthma but also in COPD. Although the evidence about Type2 biomarkers in COPD is inconsistent and less robust, Type2 biomarkers have shown some potential when analyzing various clinical outcomes or therapeutic response to ICS. In this article, we review the existent and emerging Type2 biomarkers with clinically higher applicability in the management of COPD.

## 1. Introduction

Chronic obstructive pulmonary disease (COPD) is heterogeneous in the underlying pathophysiology of airway inflammation and its response to anti-inflammatory agents, despite being characterized by persistent respiratory symptoms and airflow limitation [1]. Although airway inflammation in COPD is typically thought to be driven by Type1 immune responses, Type2 airway inflammation appears to underlie the disease in some patients in a stable state and during exacerbations [2]. A previous study demonstrated that some COPD patients showed the gene expression of Type2 inflammation in the airway and this subset was associated with inhaled corticosteroid (ICS) response [3].

Interestingly, the relationship between COPD and diseases associated with Type2 inflammation from the perspective of impaired lung development is increasingly highlighted by recent epidemiologic studies on the origin of COPD [4,5,6,7,8,9,10,11,12]. Given such a background, biomarkers reflecting Type2 airway inflammation may play an essential role in predicting the disease activity (i.e., predicting future exacerbations or the rate of lung function decline) and achieving medicinal precision for COPD. In fact, there has been a large number of studies about the possible role of Type2 biomarkers in COPD in the last few years, so that the prevalence and meaning of existent Type2 biomarkers such as sputum and blood eosinophils, exhaled nitric oxide fraction (FeNO), immunoglobulin E (IgE), and atopy have attracted attention in patients with COPD. Additionally, not only are Type2 biomarkers used in daily clinical practice, but emerging type2 biomarkers are also being searched for. In this article, we discuss existent and emerging Type2 biomarkers with clinically higher applicability in the management of COPD.

## 2. Type2 Airway Inflammation in COPD

The pathogenesis of COPD is closely associated with cigarette smoking [13]. As a result, it is characterized by increased numbers of macrophages in the peripheral airway, lung parenchyma, and pulmonary vessels, together with increased activated neutrophils and increased lymphocytes that include Type 1 CD8^+^ T (Tc1) cells, Type 1 T helper (Th1) cells, Type 17 T helper (Th17) cells, and Group 3 innate lymphoid cells (ILC3) [14]. However, in some patients, there may also be an increase in eosinophils, Type 2 T helper (Th2) cells, or Group 2 innate lymphoid cells (ILC2). Around 10–40% of COPD patients demonstrate increased eosinophilic inflammation in the sputum and or blood [15,16,17] with increased Type2-transcriptome signatures [3].

Eosinophilic airway inflammation derived from innate and adaptive immune responses is well described for asthma. Th2 lymphocytes have crucial roles in orchestrating adaptive immune responses. Allergens, presented to naïve CD4+ T cells by dendritic cells, induce differentiation toward Th2 cells, which produce interleukin (IL)-4, IL-5, and IL-13 cytokines, leading to IgE class switch in B cells, airway eosinophilia, and mucous hypersecretion [18]. On the other hand, in innate immune responses, epithelium-derived cytokines (IL-25, IL-33, thymic stromal lymphopoietin [TSLP]) are released in response to air pollutants, microbes, or glycolipids. These bind to receptors on ILC2s, activating them to produce the Th2-associated cytokines IL-5 and IL-13, which lead to eosinophilia, mucous hypersecretion, and airway hyperresponsiveness [18].

Although the mechanism of Type2 airway inflammation in COPD remains to be determined, as with Th1-mediated COPD, Type2 inflammation in COPD is likely to be a combination of innate and adaptive immunity. In particular, the role of ILC2s, which might be necessary for intrinsic asthma, but also exists in COPD, has recently been attracting attention [19,20]. ILC2s can be regulated by epithelial mediators released as a result of epithelial cell injury resulting from cigarette smoke and viral infection. In a mouse model of cigarette smoke-induced COPD, the expression of both IL-33 and TSLP was increased in lungs [21,22,23,24]. The upstream cytokines of TSLP are secreted from epithelial cells of COPD patients [25] and increased TSLP expression has been shown in the airway smooth muscle cells of patients with COPD [26]. IL-33 expression is also increased in epithelial progenitor cells of COPD patients and is associated with increased expression of IL-13 and the mucin 5AC gene [21]. Moreover, IL-33 in serum as well as in exhaled breath condensate was increased and correlated with the disease severity and increased blood eosinophil count in patients with COPD [27,28,29,30]. Although whether innate Type2 immune responses are involved in the pathogenesis of COPD, or whether other mechanisms are driving type2 inflammation in COPD remains unclear, these findings suggest such involvement, especially in patients with Type2-high COPD.

As well as in the stable state, a diversity of airway inflammation is also observed in COPD exacerbations. Papi et al. evaluated the relationship among pathogens identified in COPD exacerbations and specific airway inflammation patterns [31]. They found that viral and/or bacterial infection was detected in 78% of exacerbations: viruses in 48% and bacteria in 55%. Moreover, airway eosinophilia was related to exacerbations of viral infections. Other studies reported that eosinophilic airway inflammation was observed in 28% of exacerbations [32], and the degree of eosinophilia shown during exacerbations was higher than that seen in a stable state [33,34]. On the other hand, a recent study found that the blood eosinophil levels during exacerbations were lower than during a stable state [35]. This result may be explained by a report describing that the blood eosinophil counts decreased due to the influence of the bacterial load at the time of exacerbation compared with the stable state [36]. These inconsistent results may be due to the heterogeneity of airway inflammation in the COPD exacerbations.

## 3. Relationship between COPD and Type2 Inflammation from the Perspective of Impaired Lung Development

The controversy that asthma and COPD are not always separate diseases began with the Dutch Hypothesis in 1961 [37], and there has been a growing appreciation over the years. Nowadays, Asthma-COPD overlap (ACO) has been identified in clinical practice by the features that it shares with both asthma and COPD. Of note, the relationship between COPD and diseases associated with Type2 inflammation is highlighted by recent epidemiologic studies on the origin of COPD [4,5,6,7,8,9,10,11,12]. In the Tasmanian Longitudinal Health Study, 8583 participants were followed from 7 to 53 years of age with spirometry at 7, 13, 18, 45, 50, and 53 years [12]. This study found that not only personal smoking but also childhood asthma, allergic rhinitis, eczema, bronchitis, pneumonia, and maternal smoking were significant risk factors for reduced pulmonary function. Moreover, if the forced expiratory volume in 1 s (FEV_1_) was lower than average early in life, the decline accelerated. The group had a high rate of childhood asthma (37%), and 46% of the subjects in the group developed COPD at 53 years of age. Because childhood asthma, allergic rhinitis, and eczema are well associated with Type2 inflammation, this study clearly suggests the involvement of Type2 inflammation in the pathogenesis of COPD. In this context, the importance of Type2 biomarkers in COPD may increase.

## 4. Existent Type2 Biomarkers in COPD

### 4.1. Sputum Eosinophils

Sputum eosinophilia in asthmatics is strongly associated with a good response to corticosteroid therapy, and tailored strategies aimed to normalize sputum eosinophils reduce the exacerbation frequency and severity [17]. Sputum eosinophilia is occasionally seen in COPD patients in a stable state and during exacerbations [16,38,39,40]. Studies have used a threshold of 2% or 3% in sputum for defining eosinophilic airway inflammation in COPD [16,32,39,41]. Sputum eosinophilia in COPD patients in a clinically stable state predicts a risk of future exacerbations and is associated with a favorable response to ICS [16,39,41,42,43]. Moreover, a study from the SPIROMICS cohort (*n* = 2737) found that elevated sputum eosinophils had significant associations with multiple measures of COPD severity, including exacerbations, airflow limitation, and worse quality of life [44]. Although induced sputum is less invasive than tissue biopsy, it is somewhat difficult to perform and not available in every clinical setting. Furthermore, sputum induction can sometimes cause airway constriction. Due to these drawbacks, the induction of sputum eosinophils, although highly useful, is not widely used in clinical practice.

### 4.2. Blood Eosinophils

#### 4.2.1. Blood Eosinophil Levels in COPD and Modifying Factors

The blood eosinophils levels have been proposed as a surrogate marker of sputum eosinophils, which has the weaknesses referred to in the previous section but is widely used for both asthma and in COPD. The blood eosinophil level in asthmatics has a well-established correlation with sputum eosinophils, and serves as a biomarker for future exacerbations, a decline in lung functions, and the response to ICS and biologics [45,46,47,48,49,50]. On the other hand, in COPD patients, the correlation between contemporaneous blood eosinophils and sputum eosinophils has been discussed, and discordances have been noticed between sputum, tissue, and blood eosinophil counts [44,51,52,53,54]. In a recent study, blood and sputum eosinophils in COPD patients did not correlate as well as in patients with asthma [49]. Interestingly, in elderly subjects and patients with comorbidities such as hypertension, ischemic heart disease, and atrial fibrillation, no correlation between blood and sputum eosinophils was found. Since most COPD patients have those characteristics and comorbidities, this finding is very important when considering blood eosinophils as a biomarker in clinical practice.

Although there are challenges to using blood eosinophils as a surrogate marker of sputum eosinophils, many studies have investigated the blood eosinophil levels in COPD patients because of their simplicity. The prevalence of blood eosinophilia in patients with COPD varies depending on the study population and threshold used for evaluation. In the Copenhagen General Population Study (*n* = 7225), 23% of patients had blood eosinophil counts ≥ 280 cells/μL [55]. In a post-hoc analysis of the WISDOM trial (*n* = 2420), 53% of patients had ≥ 150 eosinophil cells/µL, 20% had ≥300 eosinophil cells/µL, and 11% had ≥ 400 eosinophil cells/µL [56]. In the Hokkaido COPD cohort study, excluding asthma carefully by respiratory specialists, 19% of the patients had blood eosinophil counts ≥300 cells/μL [57].

Another critical point is that the stability and reproducibility of blood eosinophil measurements have also been debated [16,35,58,59]. Data on two large cohorts of COPD patients revealed that, in three measurements of blood eosinophils measured over two years, there was considerable variability in blood eosinophils, with only 15% of patients showing a persistently high blood eosinophil count (>300 cells/μL) [59]. This is because, regardless of the airway inflammation, the blood eosinophils are affected by various factors such as drugs, malignant tumors, and parasitic infections [60], so a single measurement of blood eosinophils may not be reliable. For example, a previous study using a single measurement found that, in stable COPD patients, the optimal cut-off value for blood eosinophils, which reflects sputum eosinophils ≥3%, was 215 cells/μL (area under the curve [AUC] 0.76, sensitivity 60% and specificity 93%) [61]. However, in another study, only half of the patients with blood eosinophils of 215 or greater had sputum eosinophils ≥3% [49]. From the aspect of influential factors, stability, and reproducibility, more systematic studies of blood eosinophils in COPD patients are needed to establish surrogate markers that reliably reflect the airway inflammation.

#### 4.2.2. Blood Eosinophils as a Predictor for COPD Exacerbations

It is clear that elevated blood eosinophil counts are a risk factor for future exacerbations in patients with asthma [62]. In the field of COPD, several studies have investigated whether the blood eosinophils can be used to predict future exacerbations. The results have varied, with both negative and positive findings reported [15,44,54,55,57,59,63,64,65,66,67]. In a prospective analysis from the Copenhagen General Population Study, higher blood eosinophil levels during stable phase (>340 cells/μL) were associated with a 1.76-fold increased risk of severe exacerbations [55]. A historical population study suggested that patients with elevated blood eosinophil levels (>450 cells/μL) had a 13% higher exacerbation rate during the following year than those with lower levels, with the findings being most clearly demonstrated in patients who were ex-smokers [66]. A recent publication showing both cross-sectional and prospective data from COPDGene and ECLIPSE described that higher blood eosinophil levels (>300 cells/μL) were associated with increased exacerbation risk, the incidence rate ratios of which were 1.32 and 1.22, respectively [15]. Especially, the findings were most clearly found in patients with a history of frequent exacerbations. These studies, which showed an association between blood eosinophils and frequency of exacerbations, should take into account the large proportion of patients with a history of frequent exacerbations.

On the other hand, several studies showed that the exacerbation rate of COPD patients was not related to blood eosinophil levels [44,54,57,59,64,65,67]. In these studies, there was no association even when examined with different cut-off values at a cut-off of ≥200 or ≥300 cells/μL, or ≥2%, ≥3%, or ≥4%. Of note, Casanova et al. investigated the prevalence and stability of a high level of blood eosinophils (≥300 cells/μL) and its relationship to future exacerbations by using the data from two large cohorts of COPD patients [59]. They found that, in three measurements of blood eosinophils measured over two years, there was notable variability in the levels of blood eosinophils. Blood eosinophil levels ≥300 cells/μL persisting over two years were not a predictor for COPD exacerbations. Also, data from the SPIROMICS cohort demonstrated that the blood eosinophil level as a single biomarker did not accurately predict sputum eosinophils, and did not show an association with exacerbations unless observed in the background of increased sputum eosinophils [44]. Thus, much remains to be done to utilize the blood eosinophils as a predictor of exacerbations in COPD patients.

#### 4.2.3. Relationship between Lung Function and Blood Eosinophils in COPD

It is well known that the blood eosinophils are strictly related to the lung function in adult asthma, and blood eosinophils are associated with airflow obstruction and enhanced decline in lung function [68]. However, only a small number of studies have investigated the influence of blood eosinophils on the decline in lung function. The Hokkaido COPD Cohort Study Group investigators reported that patients with a rapid decline in FEV_1_ displayed lower levels of blood eosinophils [69,70]. Moreover, they suggested that patients with elevated blood eosinophils (≥300 cells/μL) had significantly slower annual FEV_1_ decline [57]. In contrast, the ECLIPSE investigators found no significant differences in the rate of decline in FEV_1_ according to the blood eosinophil pattern using a cut-off value of 2% [16]. This finding was similar to that in another study using a cut-off value of 300 cells/μL [67]. Although further large cohort longitudinal studies are needed, the effect of blood eosinophils on respiratory function in COPD patients may not be as strong as that in asthma patients.

#### 4.2.4. Blood Eosinophils as a Biomarker of ICS Treatment Response in COPD

From the viewpoint of the effectiveness and the risk of adverse effects such as pneumonia and osteoporosis [71,72], there is a need to identify which patients will benefit from ICS. A number of studies have investigated whether the blood eosinophil levels can be used to predict the effectiveness of ICS. In particular, many studies examined whether blood eosinophil levels can be used to predict whether patients will benefit from the prevention of future exacerbations by add-on ICS therapy in combination with long-acting β2-agonist (LABA) or LABA/long-acting muscarinic antagonist (LAMA) compared to bronchodilators alone [73,74,75,76,77,78,79,80,81]. Judging from these reports, the Global Initiative for Chronic Obstructive Lung Disease (GOLD) statements have recommended the use of ICS in combination with LABA or LABA/LAMA in patients with frequent exacerbations and blood eosinophil counts ≥300 cells/μL, and is considering the use of ICS in combination with LABA or LABA/LAMA when blood eosinophil counts are between 100 and 300 cells/μL, while ICS is not at all recommended if the blood eosinophil counts are <100 cells/μL [14].

A few studies have investigated the usefulness of blood eosinophils to predict the risk of exacerbations after ICS withdrawal from triple therapy (ICS/LABA/LAMA) [56,82]. Similar results were obtained, and only patients with baseline elevated blood eosinophil counts (≥300 or 400 cells/μL) were at an increased risk of exacerbations compared with patients with lower eosinophil counts.

In contrast, several observational studies among COPD patients using ICS did not find an association between blood eosinophilia and reduced risk of exacerbations [83,84]. The lack of relationship shown in the observational studies suggests that this association may not be present within the real-world COPD population. Although randomized controlled trials (RCTs) address important findings for us, we need to keep in mind that it has been estimated that the patients with COPD selected for RCTs are representative of about 7% of the entire COPD population [85].

In a real-world survey, most COPD patients belonged to GOLD A or B, who are less likely to experience exacerbations but continue to have daily symptoms [86,87]. Therefore, knowledge concerning the effects of ICS other than preventing exacerbations, such as increased lung function and improvement of symptoms, are also required. A few studies have sought to determine whether the blood eosinophil levels can predict which patients will benefit in terms of lung function and/or symptoms from add-on ICS therapy. In a post-hoc analysis of the ISOLDE study, which compared an ICS with placebo, patients were also stratified using a baseline blood eosinophil threshold of 2% [75]. This study found that a baseline blood eosinophils ≥ 2% identified a group of patients with slower rates of decline in FEV_1_ when treated with ICS. Recently, we reported a prospective study to detect Type2 biomarkers for predicting short-term improvements in both quality of life (QOL) and airflow limitation by ICS in 43 symptomatic COPD patients who had been taking bronchodilators (De-stress study) [88]. The study excluded subjects who were current smokers with concomitant asthma. Seventy percent of the patients took LAMA/LABA, and 90% of the patients were classified as GOLD B. After 12 weeks of ICS treatment, 28% of the patients showed significant improvement in the COPD assessment test (CAT) and FEV_1_. Among the several Type2 biomarkers, the absolute blood eosinophil counts and the percentage of blood eosinophils were less likely to predict the efficacy of ICS compared to FeNO. Nonetheless, our results did not exclude the possibility of blood eosinophils being used to predict the effects of ICS in COPD. In fact, more than 60% of patients with blood eosinophilia (≥300 cells/μL) showed favorable effects from ICS in the present study. Due to the discrepancies reported in various studies, further studies are needed to determine whether blood eosinophils are useful as a biomarker for determining the use of ICS.

### 4.3. FeNO

#### 4.3.1. FeNO Levels in COPD and Modifying Factors

FeNO has been established as a useful biomarker of Type2 airway inflammation and a guide for anti-inflammatory therapy in asthma [89,90,91]. Currently, FeNO values can be determined noninvasively, reproducibly, and easily measured in close to real-time using portable analyzers. Therefore, the Global Initiative for Asthma recommends that FeNO should be used as part of the clinical assessment in asthma [92]. Nitric oxide is produced mainly by inducible nitric oxide synthetase (iNOS) in the epithelial cells of the bronchial wall in response to IL-4 and IL-13 via the signal transducer and activator of transcription 6 pathway [93]. Since FeNO is a surrogate biomarker for Type2-high asthma, particularly a downstream molecule of IL-4/13, several trials to apply FeNO to a biomarker to predict the efficacy of asthma drugs targeting IL-4/13 have been performed [94,95].

The role of FeNO in COPD remains controversial. Although iNOS is highly expressed in COPD patients [96,97,98], the results of studies of the FeNO levels in COPD are contradictory, showing elevated, similar, and reduced levels. According to a recent systematic review and metanalysis, FeNO levels varied widely among several studies (I^2^ = 96%) and were mildly elevated in patients with stable COPD compared with healthy controls (standard mean difference [SMD] 1.21, 95% confidence interval [CI] 0.47–1.96) [99]. Additionally, it was found that the FeNO levels were much higher in ex-smokers than in current smokers (SMD 2.05, 95% CI 1.13–2.97). This heterogeneity can be explained by various factors. It is known that current smoking reduces the FeNO levels through reduced production and increased consumption of NO [100,101,102]. In addition, ICS treatment decreases the FeNO levels [88,103,104,105]. FeNO is also influenced by comorbidities such as atopy and rhinitis [101,102].

There are many studies that compared FeNO in COPD subjects with ACO [106,107,108,109,110,111,112,113]. Although higher levels of FeNO were observed in ACO patients compared to those with COPD-only, it is still difficult to interpret the results of these studies. These studies used not only different cut-off values but also different definitions of ACO. Meanwhile, Tamada et al. investigated 331 COPD patients for asthma-like airway inflammation or atopic factors using FeNO and serum IgE, respectively [112]. In this study, the values of FeNO were shown in detail, even the histogram. The prevalence rate of FeNO >25 ppb was 36.9%, those of >35 ppb, >50 ppb were 16.3%, and 5.1%, respectively. Similarly, another recent study reported that, in 178 severe (FEV_1_ predicted ≤ 50%) COPD patients, FeNO ≥ 25 ppb was found in 32.9%, and FeNO ≥ 50 ppb was 2.6% [113].

#### 4.3.2. Relationship to COPD Exacerbations in FeNO

Several studies have addressed the relationship between COPD exacerbations and FeNO. In the changes of the FeNO levels, most studies reported that FeNO significantly increased during exacerbations [31,114,115,116,117,118]. High FeNO values in hospitalized COPD exacerbation patients returned to the control values only months after these steroid-treated patients were discharged [116]. Rhinovirus infections induce increases in the FeNO levels as a result of upregulated iNOS expression in the airway epithelium [119]. Viral infections are a major cause of COPD exacerbations [120]. Thus, the elevation of FeNO in COPD exacerbations may be the direct result of viral infection.

Fewer studies have examined whether FeNO is a predictive marker for COPD exacerbations. A recent prospective study showed that persistently elevated FeNO levels (≥20 ppb) in stable COPD patients were associated with a significantly higher risk of exacerbations [121]. After adjusting for potential confounding variables, the hazard ratio for exacerbations was higher in the latter group (1.579 [95% CI 1.049–2.378], *p* = 0.029). Moreover, the time to first moderate and then severe exacerbations was shorter in patients with persistently high FeNO.

#### 4.3.3. Relationship between Lung Function and FeNO in COPD

High FeNO levels in severe asthma could be used to identify patients with the greatest airflow limitation [122]. Previous prospective studies in asthmatics showed that higher levels of FeNO were associated with a rapid decline in lung function [123]. IL-4/IL-13 pathways facilitate airway smooth muscle contraction and proliferation, and goblet cell hyperplasia, mucus production, increased extracellular matrix secretion by fibroblasts, and subepithelial basal membrane thickening, all of which are features of airway obstruction [124]. The roles of IL-4 and IL-13 in orchestrating the pathogenesis of asthma may help to explain the relationship between high FeNO levels and airflow limitation. However, the relationship between lung function and FeNO in COPD is not well understood. Most studies reported no association between the FeNO levels and pulmonary function [104,108,112,125,126,127], while two studies showed an association. One study found a negative correlation between FEV_1_/forced vital capacity (FVC) ratio and the FeNO levels in patients with COPD (r = −0.59, *p* = 0.028) [128]. Another study showed that there was a negative correlation between the FeNO values in COPD and FEV_1_ (r = −0.50, *p* = 0.004) [129]. In contrast to previous findings, it has been reported that there was a higher proportion of patients with severe airflow limitation in the low FeNO group (<25 ppb) compared with the high FeNO group [130]. This discrepancy may be derived from the patients’ heterogeneity, such as age and treatment, and differences in the cut-off values of FeNO in each study.

#### 4.3.4. FeNO as a Biomarker of the ICS Treatment Response in COPD

FeNO might be a predictive biomarker of the response to ICS treatment in COPD as in asthma. A recent systematic review and meta-analysis investigated the response of the FeNO levels to ICS treatment in COPD patients [131]. Five studies of 171 patients were included in the analysis. Two-thirds were ex-smokers, and this analysis excluded studies with patients having a diagnosis of asthma. There was a significant decrease of FeNO in ex-smoking COPD patients following ICS treatment (−7.51, 95% CI: −11.51 to −3.51; *p* = 0.003), and in a population of subjects that included both smokers and ex-smokers. However, this analysis focused on the changes of FeNO as a treatment response so it was not possible to analyze the association of FeNO levels with QOL, pulmonary function, or reduction in exacerbations. Some studies found an inverse relationship between FeNO levels and FEV_1_ in ex-smokers but not in smokers with COPD [115,132,133].

Several studies demonstrated that FeNO predicted the response when adding corticosteroids. Elevated FeNO in COPD patients may also be a variable signal for an increased spirometric response to systemic corticosteroids [125]. Other studies reported that FeNO could be a predictor of increased FEV_1_ by ICS [104,105,133]. However, whether FeNO is useful for predicting both symptoms and airflow limitation with ICS treatment in patients with COPD is poorly documented. The findings of our de-stress study demonstrated that, in the several Type2 biomarkers, FeNO was identified as the most accurate predictor for benefits from ICS (AUC = 0.92) [88]. Furthermore, the baseline FeNO values were significantly correlated with changes in FEV_1_ (*ρ* = 0.835, *p* < 0.0001) and CAT (*ρ* = −0.672, *p* < 0.0001) after treatment with ICS, supporting the usefulness of FeNO as a predictor of ICS responsiveness. We proposed two cut-off values for FeNO: 35 ppb is associated with certainty for response inclusion, and 20 ppb is associated with certainty for response exclusion. However, further studies are needed to confirm the usefulness of FeNO for many patients, including current smokers, and determine the appropriate cut-off value. Furthermore, future prospective studies should be focused on detecting biomarkers for predicting the long-term prevention of exacerbations.

### 4.4. IgE, Atopy

#### 4.4.1. Evaluation of Atopy in Patients with COPD

Atopy is defined as “a personal, and/or familial tendency, to become sensitized and produce IgE antibodies in response to ordinary exposure to allergens, usually proteins” [134]. There are various measures of atopy, such as positive skin-prick tests and elevated serum IgE levels. Current studies define atopic sensitization as a positive allergen-specific serum IgE (most commonly specific IgE levels >0.35 kUa/L) or a positive skin-prick test (usually, but not exclusively, a wheal diameter ≥3 mm) to any typical food or inhalant allergen [135]. Atopy has been recognized as a significant contributor to the pathophysiology of asthma. Although atopy has been less well studied in patients with COPD, it has been considered that atopy is a risk indicator of COPD since more than 50 years ago. The Dutch Hypothesis, put forward in 1961, proposes that there are common host factors for asthma and COPD, including atopy and airway hyperresponsiveness [136]. In a longitudinal study, there was a significant inverse association between total serum IgE and FEV_1_/FVC that was independent of smoking and asthma status [137]. These findings suggest that atopy could potentially influence the impairment of lung growth and decline in lung function over time. From the findings of longitudinal studies, it continues to become clearer that atopy has a potential role in the disease expression or progression of COPD. Therefore, the evaluation of atopy for the management of COPD may become important in the future.

The reported positive rate of atopy in COPD varies between about 15–40% [57,112,138,139,140,141,142,143,144,145]. Jamieson et al. found a prevalence of 29% with atopy in a population of 77 former smokers with COPD in the CODE cohort. They found that COPD patients with allergic sensitization had increased respiratory symptoms and exacerbation rates [142]. Fattahi et al. demonstrated the atopic status in the EUROSCOP [141]. It was also evaluated for the incidence and remission of respiratory symptoms of patients during a 3-year follow-up and for the association of atopy with the decline of lung function. This study found that atopy, defined as a positive specific IgE, was present in 18% of the 1277 current smoking COPD patients. It was found that, compared with nonatopic COPD patients, atopic COPD patients were more likely male, younger, and obese. Moreover, the presence of atopy was associated with increased cough and chest tightness, but not with a decline of FEV_1_. In contrast, other studies showed an association with the sensitization to Aspergillus antigens and pulmonary function. Bafadhel et al. determined that atopy (defined by a positive skin prick test and/or elevated allergen-specific antibodies) was present in 34% of 128 COPD patients [140]. Especially, sensitization to *Aspergillus fumigatus* was shown to be 13%, which was associated with severe airflow limitation. Jin et al. reported that the prevalence of elevated total-IgE and sensitization to *Aspergillus fumigatus* was 47% and 15%, respectively [139]. In this study, total serum IgE levels were found to be negatively correlated with FEV_1_% predicted. Given the multiple links between *Aspergillus* species and bronchiectasis [146], sensitization to Aspergillus antigens may play an important role in the development of COPD-related bronchiectasis. In fact, a recent report found a high prevalence of *Aspergillus fumigatus* sensitization in COPD patients (18%), which highlights a potential role for sensitization to *Aspergillus fumigatus* in COPD-related bronchiectasis [138].

#### 4.4.2. Atopy as a Biomarker of ICS Treatment Response in COPD

There has been a growing interest in finding the link between atopy and responses to ICS therapy in COPD patients. However, only a few studies evaluated whether IgE/atopy can predict the response when adding corticosteroids in COPD patients. In the EUROSCOP, compared to non-atopic COPD patients, those with atopy more often showed remission of respiratory symptoms when treated with ICS [141]. However, there was no significant difference in changes in post-bronchodilator FEV_1_ between atopic and non-atopic patients who received ICS. Akamatsu et al. examined the possibility of whether atopy predicts the ICS/LABA treatment response in COPD patients. The patients with atopy (defined by positive specific IgE) showed significantly higher improvement in FEV_1_ [147], and atopy yielded 60% sensitivity and 89% specificity for an improvement in FEV_1_. The findings of our de-stress study were in line with previous studies. Atopy was useful for predicting improvements in both symptoms and airflow limitation with ICS treatment [88]. AUC for atopy was 0.79, and the sensitivity and specificity yielded 75% and 84%, respectively.

### 4.5. Composite Biomarkers

In recent years, the diversity of airway inflammation and the development of biologics for asthma have led to increased attention given to evaluations of the composite Type2 biomarkers. Some researchers have suggested the importance of suppressing both persistent eosinophilia and high FeNO for the management of asthma control [50,148]. Recently, the diversity of airway inflammation in COPD has also attracted attention. Based on the findings that the correlations between FeNO values and blood eosinophil counts in patients with severe and extremely severe COPD were not significant, Chen et al. have proposed that FeNO and blood eosinophil counts should be both evaluated in patients with COPD [113]. The use of composite Type2 biomarkers has been recommended for the definition and diagnosis of ACO in several national and international guidelines [149,150]. In fact, several studies showed that the combination of blood eosinophil counts and FeNO was useful in differentiating asthma from COPD or ACO from COPD [151,152].

**Figure 1:**
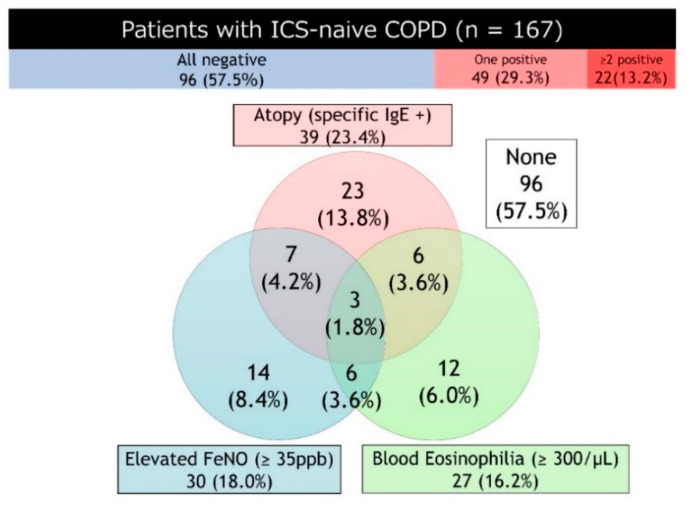
Venn diagram and the positive prevalence of Type2 inflammation features. Abbreviations: COPD, chronic obstructive pulmonary disease; FeNO, exhaled nitric oxide fraction (FeNO); ICS, inhaled corticosteroid; IgE, Immunoglobulin E.

Additionally, in clinical practice, it could be more important for predicting the therapeutic effect of ICS to evaluate the composite Type2 biomarkers. Akamatsu et al. reported that combining FeNO and specific IgE may be a surrogate marker for predicting the response to ICS/LABA on airflow limitation in patients with COPD [147]. For these reasons, we predict that evaluating the composite Type2 biomarkers in COPD patients will become increasingly important. Thus, we focused on the prevalence of Type2 inflammation features of 167 ICS-naïve COPD patients using a combination of multiple Type2 biomarkers available for daily clinical practice. In this study, COPD outpatients were retrospectively enrolled in two tertiary care facilities in Japan from April 2017 to March 2020. Patients with current diagnosis of asthma and current smokers were excluded. The Type2 inflammation features were determined by the presence of atopy (positive specific IgE for any inhaled antigen) and/or elevated FeNO (≥35 ppb), and/or blood eosinophilia (≥300 cells/μL). Since the Japanese Respiratory Society guidelines recommend that 35 ppb as a reference value to capture the inflammatory condition characteristic of asthma, we have set the reference value for FeNO at 35 ppb [153,154]. A Venn diagram and the positive prevalence of Type2 inflammation features are shown in Figure 1. Twenty-three percent of subjects had atopy, 18% of those had elevated FeNO, and 16% of those had blood eosinophilia. By combing Type2 biomarkers, more than 40% of the patients were positive for at least one Type2 biomarker, and 13% of the patients had multiple biomarkers. Furthermore, patients with multiple positive biomarkers showed a trend in higher rates of exacerbation than patients without them. Although the role of the composite Type2 biomarkers in COPD patients remains unclear, the findings of our study will help develop a clinical decision-making strategy for the appropriate use of ICS in COPD patients.

## 5. Emerging Type2 Biomarkers in COPD

### 5.1. Periostin

Periostin, a matricellular protein produced by airway epithelial cells under the control of IL-4 and IL-13, is a key molecule linking TType2/eosinophilic airway inflammation and remodeling in asthma [155]. Periostin can be detected in the blood and is relatively stable without being affected by ICSs, serving as a marker of the phenotype and a rapid decline of pulmonary function [156]. When interpreting data, attention should be paid to differences in the ELISA kits used. An open-label, single-arm, prospective study in 130 stable COPD patients found that high plasma periostin (Adipo Bioscience kit: >23 ng/mL) levels were associated with FEV_1_ responders (>12% and >200 mL increase in FEV_1_ from baseline: 43% of responders versus 24% of non-responders, *p* = 0.027) after 12-week treatment with an ICS/LABA [157]. However, serum periostin alone was not a significant predictor of FEV_1_ responders. The serum periostin (Human Periostin/OSF-2 DuoSet kit) levels in 155 patients hospitalized for acute exacerbations of COPD were higher on admission compared to discharge (34.7 versus 25.9 ng/mL, *p* = 0.003), and frequent exacerbators had higher levels of serum periostin at discharge. However, there were no correlations between the serum periostin levels and severity of airflow obstruction or blood eosinophils on admission [158]. A previous study assessed the effect of smoking on serum periostin by using the Elecsys Periostin immunoassay in COPD patients and healthy controls [159]. COPD smokers and past-smokers had significantly higher periostin levels (51.8 and 54.8 ng/mL, respectively) compared to healthy smokers (44.6 ng/mL), but not healthy never smokers (49.7 ng/mL). However, the periostin levels did not reflect Type2-driven inflammation, airway remodeling, or ICS treatment responsiveness.

### 5.2. Chitinase-3-Like Protein 1 (YKL-40)

Chitinase-3-like protein 1, also known as YKL-40, is a secreted glycoprotein produced by various cell types, including macrophages, neutrophils, and airway epithelial cells [160]. The multicenter BIOAIR study found that the serum YKL-40 levels were elevated in patients with asthma and COPD (the COPD levels were higher than those in asthma) compared to healthy controls [161]. Negative correlations were observed with lung function, but not with Type2 biomarkers, including FeNO, blood eosinophils, periostin, and IgE. A previous study in severe asthma patients revealed correlations between YKL-40 levels and markers associated with neutrophilic airway inflammation [162]. Another study found that serum YKL-40 levels were elevated in healthy smokers and COPD patients compared to healthy never smokers [163]. In sputum, the YKL-40 levels were increased in COPD patients compared to healthy never smokers, suggesting smoking-related activation of airway inflammatory cells. However, no significant differences were observed in the serum and sputum YKL-40 levels between patients with and those without sputum eosinophilia (>3%). Thus, it is unlikely that the serum YKL-40 levels reflect Type2/eosinophilic inflammation in COPD.

### 5.3. Eosinophil-Derived Neurotoxin (EDN)

In addition to eosinophil cationic protein and major basic protein, EDN is a granule contained in the matrix of eosinophils [164]. Previous studies indicated that serum EDN is a marker of eosinophilic inflammation [164,165,166]. One study found a significant negative correlation between the serum EDN levels and lung function (FEV_1_ and FEV_1_/FVC) in asthma patients [164]. Another study showed that serum EDN predicted the severe asthma phenotype in a multivariate regression analysis [165]. Recently, serum EDN was found to better reflect the asthma control status than blood eosinophil counts [166]. However, there are no reports assessing serum EDN as a biomarker of Type2/eosinophilic inflammation in COPD.

### 5.4. Role of Biomarkers in Identifying Asthma-COPD Overlap (ACO)

Distinguishing asthma from COPD, and diagnosing ACO as having both features, are important, although there is currently no consensus on its definition. ACO is heterogeneous and includes different phenotypes such as non-Type2 or non-eosinophilic asthma, and non-emphysematous or frequently exacerbating COPD. However, Type2 biomarkers may be useful in diagnosing ACO derived from COPD because adding an ICS to the treatment regimen is critical for such patients. Previous studies assessed the usefulness of several biomarkers in distinguishing ACO from asthma and COPD [167,168,169]. Wang et al. found that plasma YKL-40 and neutrophil gelatinase-associated lipocalin (NGAL), but not the periostin, levels, were higher in COPD patients than in patients with asthma or ACO [167]. Pérez de Llano et al. investigated the role of systemic and Type2 markers and found that serum IL-5 was lower and IL-8 was higher in COPD patients than in asthma patients [168]. However, there was no difference in these levels between COPD and ACO patients. There was also no difference in serum periostin among the three patient groups. Gon et al. reported that the serum YKL-40 levels were significantly higher in patients with ACO and COPD than in those with asthma, suggesting neutrophilic inflammation in ACO patients [169]. Together, these reports clarified the usefulness of some biomarkers in differentiating between asthma, COPD, and ACO. However, the heterogeneous nature of ACO was not considered, and the possibility of combined assessment of biomarkers was expected.

We assessed the potential roles of serum periostin, YKL-40, and EDN for identifying ACO, and investigated their relevance to other Type2 biomarkers in a cross-sectional study [170,171]. Subjects included patients with asthma (*n* = 177), ACO (*n* = 115), and COPD (*n* = 61) of the ASCOPE cohort (Nihon University Itabashi Hospital and Shizuoka General Hospital). Serum periostin, YKL-40, and EDN were measured using the Elecsys Periostin immunoassay, Human Chitinase 3-like 1 Quantikine ELISA Kit, and MBL EDN ELISA Kit, respectively. Serum periostin was significantly higher in asthma and ACO than in COPD, whereas serum YKL-40 was significantly higher in COPD and ACO than in asthma. Serum EDN was significantly higher in ACO than in asthma or COPD. Based on the cutoff values derived by a ROC analysis (periostin: 55.1 ng/mL; YKL-40: 61.3 ng/mL; and EDN: 23.0 ng/mL), patients were classified into high or low groups. The proportion of patients with high serum EDN and YKL-40 levels was significantly higher in ACO than in asthma or COPD: odds ratio, 3.85 (95% CI, 2.35–6.36); *p* < 0.001; sensitivity, 45.2%; specificity, 82.4%. The AUC of the ROC analysis for detecting ACO was significantly higher in serum EDN plus YKL-40 than in serum periostin plus YKL-40. There was a weak positive correlation between the serum periostin and eosinophil counts, FeNO, total IgE, YKL-40, FEV_1_, and FEV_1_/FVC in COPD. There was no correlation between serum YKL-40 and these parameters. There was a weak or moderate positive correlation between serum EDN and eosinophil counts, FeNO, YKL-40, FEV_1_, and FEV_1_/FVC in COPD.

**Figure 2:**
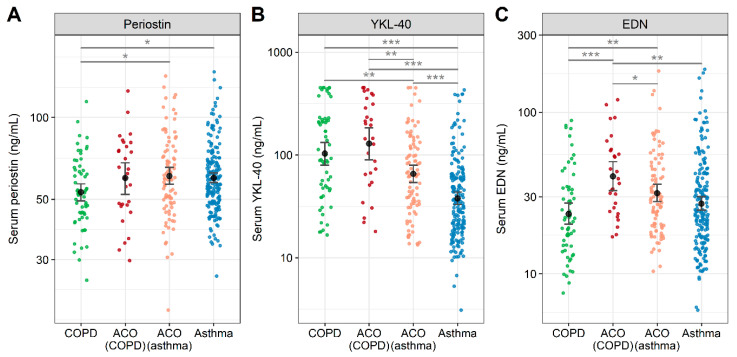
Comparison of serum periostin (**A**), YKL-40 (**B**), and EDN (**C**) among patients with COPD, ACO (COPD), ACO (asthma), and asthma. Abbreviations: ACO, asthma-COPD overlap; COPD, chronic obstructive pulmonary disease; EDN, eosinophil-derived neurotoxin; YKL-40, chitinase-3-like protein 1. * *p* < 0.05, ** *p* < 0.01, *** *p* < 0.001. Reproduced based on the data reported from Ref. [170,171].

**Figure 3:**
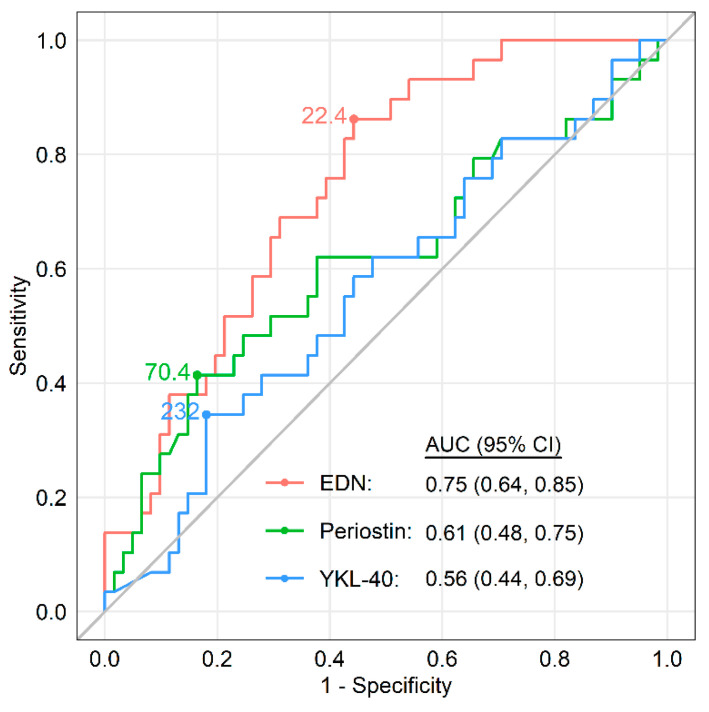
Receiver operating characteristics curves for serum EDN, periostin, and YKL-40 distinguishing ACO (COPD) from COPD with the optimal cut-off values: EDN, 22.4 ng/mL; periostin, 70.4 ng/mL; and YKL-40, 232 ng/mL. Statistical significance is shown for EDN alone. Abbreviations: ACO, asthma-COPD overlap; COPD, chronic obstructive pulmonary disease; EDN, eosinophil-derived neurotoxin; YKL-40, chitinase-3-like protein 1. Reproduced based on the data reported from Ref. [170,171].

One hundred and fifteen patients with ACO consisting of 86 patients derived from asthma, denoted as ACO (asthma), and 29 derived from COPD, denoted as ACO (COPD). Serum biomarkers in these patients are shown in Figure 2. Serum EDN levels were significantly higher in patients with ACO (COPD) than in those with COPD. Possible explanations for the difference between patients with ACO (COPD) and ACO (asthma) included lower FEV_1_/FVC (42.6% [34.7–61.4] vs. 60.7% [48.4–66.4], *p* < 0.001) and fewer ICS users (28% vs. 95%, *p* < 0.001) in ACO (COPD) patients. However, there was no difference in the serum periostin or YKL-40 levels between patients with ACO (COPD) and those with COPD. The AUC of the ROC analysis for differentiating ACO (COPD) from COPD was significantly higher in serum EDN than in serum periostin or YKL-40, with a cut-off value of 22.4 ng/mL, a sensitivity of 86.2%, a specificity of 55.7%, and an AUC of 0.75 (95% CI, 0.64–0.85; *p* = 0.002) (Figure 3). If confirmed in other populations, these findings may facilitate more accurate identification of ACO from COPD, leading to early intervention with ICSs.

## 6. Conclusions

Existent and emerging Type2 biomarkers have been investigated extensively in patients with COPD. Although the evidence about Type2 biomarkers in COPD is inconclusive compared to asthma, Type2 biomarkers have shown some potential when analyzing various clinical outcomes or therapeutic responses to ICS. New clinical trials for ICS treatment and prospective studies for predicting the future risk could enable stratification of COPD patients according to Type2 biomarkers, which might clarify this important issue. In the near future, the examination of Type2 biomarkers will be clearly one of the mainstream tools leading to personalized COPD management.
